# Linear correlation between average fluorescence intensity of green fluorescent protein and the multiplicity of infection of recombinant adenovirus

**DOI:** 10.1186/s12929-015-0137-z

**Published:** 2015-05-14

**Authors:** Yi-Chen Tsai, Tsung-Huang Tsai, Chen-Ping Chang, Shu-Fen Chen, Yen-Ming Lee, Song-Kun Shyue

**Affiliations:** Graduate School of Life Science, National Defense Medical Center, Taipei, 11490 Taiwan; Institute of Biomodical Sciences, Academia Sinica, No. 128, Sec. 2, Academia Road, Taipei, 11529 Taiwan; School of Chinese Medicine, China Medical University, Taichung, 40402 Taiwan

**Keywords:** Adenovirus, Flow cytometry, Average fluorescence intensity, Linear correlation, Promoter activity

## Abstract

**Background:**

Adenoviral vector is an efficient tool for gene transfer. Protein expression is regulated by a number of factors, but the regulation by gene copy number remains to be investigated further.

**Results:**

Assessed by flow cytometry, we demonstrated a significant linear correlation between average fluorescence intensity of green fluorescent protein (GFP) and a wide range of multiplicity of infection (MOI), spanning from 0.01 to 200. Average GFP intensity was calculated by mean fluorescence intensity (MFI) × percentage of infection (POI) (MFI × POI) and the correlation was observed in cells transduced with GFP-expressing adenoviral vector driven either by a cytomegalovirus (CMV) promoter for 3 to 6 h or by a human phosphoglycerate kinase (PGK) promoter for 18 to 24 h. Factors impacting this linear correlation include MOI of viral vector, strength of promoter driving GFP expression, cell type transduced and incubation time after gene transfer. We also found that weak GFP signals could be interfered by background signals, whereas strong GFP signals could overshot the detection limitation of the flow cytometer and resulted in a deviation from linearity which was prevented by adjusting the setting in flow cytometer. Moreover, we compared promoter strength as measured by MFI × POI and found that the relative activity of CMV promoter to PGK promoter was 20 to 47 folds in A549 cells and 32 to > 100 folds in H1299 cells.

**Conclusions:**

The linear correlation between MFI × POI and a wide range of adenoviral MOI provides an efficient method to investigate factors regulating protein expression and to estimate virus titers.

**Electronic supplementary material:**

The online version of this article (doi:10.1186/s12929-015-0137-z) contains supplementary material, which is available to authorized users.

## Background

Recombinant adenoviral vectors have been widely used for both *in vivo* and *in vitro* gene transfer. Clinically, various genetically modified adenoviral vectors have been used for therapeutic gene delivery and for vaccination. Adenoviruses have unique features, including a high transduction efficiency, a broad host range, the ability to infect both dividing and non-dividing cells, and easy to be amplified and purified to high concentrations [1,2]. However, factors impacting transduced gene expression and protein production in these vectors have not been thoroughly investigated.

Viral vector-derived protein expression is regulated by a number of factors, including the promoter used, gene copy number within the cell type transduced and the availability of cellular machinery for transcription and translation in host cells. Copy number of transduced gene is generally considered to be linearly correlated with the amount of target protein expressed. For viral vectors carrying the green fluorescent protein (GFP) the percentage of infection (POI) or mean fluorescence intensity (MFI) are considered to be linearly correlated with multiplicity of infection (MOI) under specific conditions [3-7]. However, how these correlations relate to the promoter activity, the kinetics of target protein production in different cell types and MOI have not been well examined. Moreover, the relationship of POI and MFI to average fluorescence intensity and its correlation with MOI remains to be elucidated.

The promoter used for driving target gene expression in viral vector plays a key role in deciding protein expression level. The cytomegalovirus (CMV) and phosphoglycerate kinase (PGK) promoters are frequently used for expression of exogenous proteins in most cell types. CMV early promoter is a strong promoter used for high protein expression, whereas PGK is a housekeeping gene and its promoter has a weak activity [8]. Although relative promoter strength can be determined by immunoblotting or flow cytometry, how to quantitatively compare the promoter activity in different cell types required further assessment.

To address the above questions, we used a flow cytometry-based method to investigate the correlation between average fluorescence intensity of GFP and MOI of recombinant adenovirus in A549 and H1299 cells and found a linear correlation between average GFP intensity and a wide range of MOI, between 0.01 and 200. Based on this linear correlation, we then compared the relative promoter activity of CMV and PGK in our adenoviral vector system.

## Methods

### Cell culture

A549 and H1299 lung cancer cells were maintained in RPMI 1640 (Invitrogen) containing 10% fetal bovine serum (Hyclone). The medium was supplemented with 100 U/ml penicillin-streptomycin and cells were cultured at 37°C in a humidified 5% CO_2_ atmosphere.

### Recombinant adenovirus

E1- and E3-deleted recombinant adenoviruses encoding enhanced GFP with CMV or PGK promoter, Ad-CMV-GFP or Ad-PGK-GFP, were constructed as described [9]. The virus was purified by CsCl density gradient centrifugation. Viral preparations were aliquoted and stored in storage buffer (10 mM Tris, pH 8.0, 2 mM MgCl_2_, 4% sucrose) at -80°C until use. Viral titers of stored preparations were determined by plaque-forming assay. Cells were infected with adenovirus in culture medium.

### Flow cytometry

**Figure 1:**
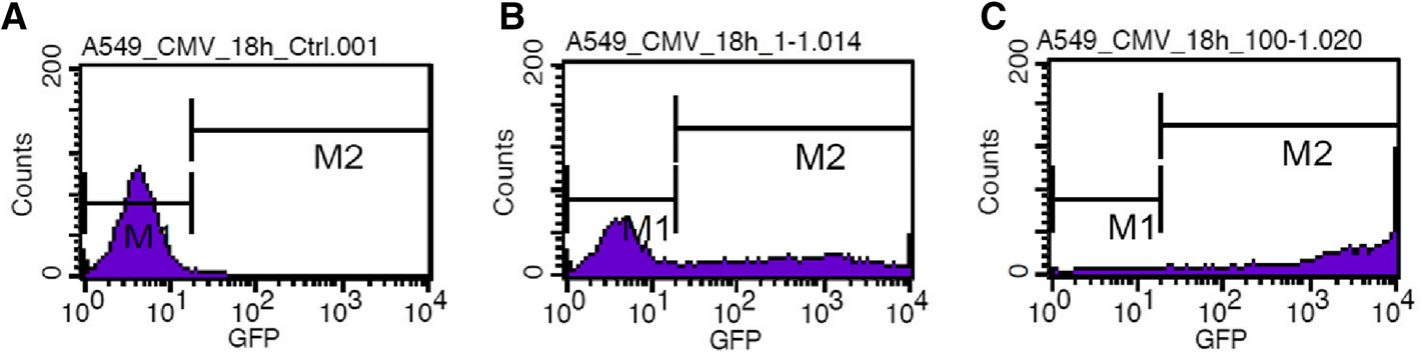
Flow cytometry of GFP intensity in A549 cells. Histogram of GFP intensity and cell count of control cells **(A)** and cells with Ad-CMV-GFP at MOI 1 for 18 h **(B)** and MOI 100 for 18 h **(C)**.

A549 and H1299 cells (1 × 10^6^) were infected with Ad-CMV-GFP or Ad-PGK-GFP at MOI 0.01 to 200 in culture medium for 3 to 48 h. Cells were collected by centrifugation, resuspended in phosphate-buffered saline and analyzed by FACScan flow cytometry (Becton Dickinson, Mountain View, CA). In total, 1 × 10^4^ cells were scanned for analysis. The fluorescence data obtained with uninfected cells was marked as the background (M1) (Figure 1A). A signal stronger than M1 was considered a positive fluorescent signal (M2) (Figure 1B). We obtained the MFI and POI for M2. Results were the average of 3 repeats. The default setting for fluorescence detection was used for most experiments. To prevent the GFP signal overshooting the detection limitation, the sensitivity of the flow cytometer was adjusted lower to extend the detection limitation as indicated.

### Quantitative real-time RT-PCR for coxsackie and adenovirus receptor transcript

Total cellular RNA was isolated by use of TRI reagent and underwent cDNA synthesis with oligo-dT and SuperScript III reverse transcriptase (Invitrogen). The cDNAs were used for quantitative real-time RT-PCR amplification with the TaqMan probe-based real-time quantification system (Applied Biosystems, Foster, CA) with the primers for coxsackie and adenovirus receptor, 5’-GAG TGG CTG ATA TCA CCA GC-3′ and 5′-CCA TCA ACG TAA CAT CTC GC-3′; and for β-actin as the internal reference, 5’-ATC CTC ACC CTG AAG TAC CC-3’ and 5’-AGC ACA GCC TGG ATA GCA AC-3’.

### Western blot analysis

A total of 30 μg cell lysates was resolved on SDS-PAGE and examined by western blot analysis as described previously [10]. β-actin was an internal control. The protein levels were quantified by densitometry (Gel Pro v.3.1, Media Cybernetics).

### Statistical analysis

The Student t test was used to compare 2 independent groups. Results are presented as mean ± SEM. A *P* < 0.05 was considered statistically significant.

### Relationship between average GFP intensity and MFI and POI

Based on flow cytometry, the mean GFP intensity and ratio of GFP-positive cells were detected as MFI and POI, respectively. The MFI was defined as the total fluorescence intensity (TFI) / number of GFP-expressing cells (Ni):

1 $$ \mathrm{M}\mathrm{F}\mathrm{I} = \mathrm{T}\mathrm{F}\mathrm{I}/\mathrm{N}\mathrm{i} $$

The POI was the ratio of GFP-expressing cells to total cell number (N):

2 $$ \mathrm{P}\mathrm{O}\mathrm{I} = \mathrm{N}\mathrm{i}/\mathrm{N} $$

From (1) and (2), we calculated average fluorescence intensity:

3 $$ \mathrm{T}\mathrm{F}\mathrm{I}/\mathrm{N} = \left(\mathrm{T}\mathrm{F}\mathrm{I}/\mathrm{N}\mathrm{i}\right)\times \left(\mathrm{N}\mathrm{i}/\mathrm{N}\right) = \mathrm{M}\mathrm{F}\mathrm{I}\times \mathrm{P}\mathrm{O}\mathrm{I} $$

Thus, average fluorescence intensity of total cells is equal to MFI × POI.

## Results

### Correlation between average GFP intensity and MOI of Ad-CMV-GFP in A549 cells

To investigate whether the average fluorescence intensity for GFP (MFI × POI) delineated in equation (3) was correlated linearly with transfected gene copy number, we used adenoviral vectors carrying the GFP gene driven either by a CMV (Ad-CMV-GFP) or a PGK (Ad-PGK-GFP) promoter to transduce A549 or H1299 cells for 3 to 48 h and examined the relationship between MFI × POI and MOI 0.01 to 200.

**Figure 2:**
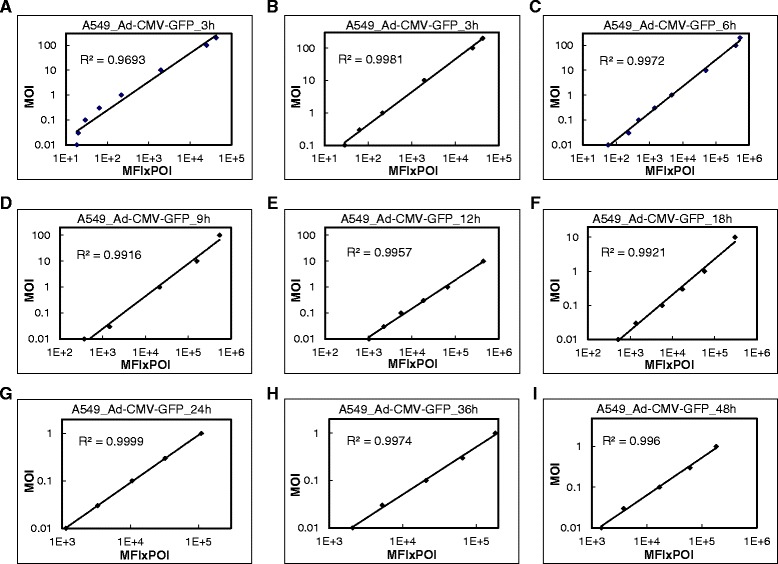
Correlation between average fluorescence intensity of GFP (MFI × POI) and MOI of Ad-CMV-GFP in A549 cells. Transduction at MOI 0.01 to 200 for 3 to 48 h for flow cytometry. **(A**-**I)** Correlation between MFI × POI and MOI. R^2^, coefficient of determination.

In A549 cells transduced with Ad-CMV-GFP, MFI × POI showed a good linear correlation with MOI (R^2^ = 0.998) for MOI 0.1 to 200 at 3 h post-infection (hpi) (Figure 2B). The MFI × POI at MOI 0.01 and 0.03 deviating from linearity could be caused by background signals interfering in the low GFP signal (Figure 2A). A good linear correlation was detected at MOI 0.01 to 200 (R^2^ = 0.997) at 6 hpi (Figure 2C). For incubation longer than 9 h, the MFI × POI with high MOI deviated from linearity (Additional file 1: Figure S1). This deviation was partially due to the limitation of the flow cytometer in that the GFP signal greater than the detection limit was counted as the maximum value and the GFP intensity was underestimated (Figure 1C). The MOI ranges for good linearity were 0.01 to 100 at 9 hpi, 0.01 to 10 at 12 and 18 hpi, and 0.01 to 1 at 24, 36 and 48 hpi for R^2^ > 0.99 (Figure 2D-I and Additional file 1: Figure S1).

**Figure 3:**
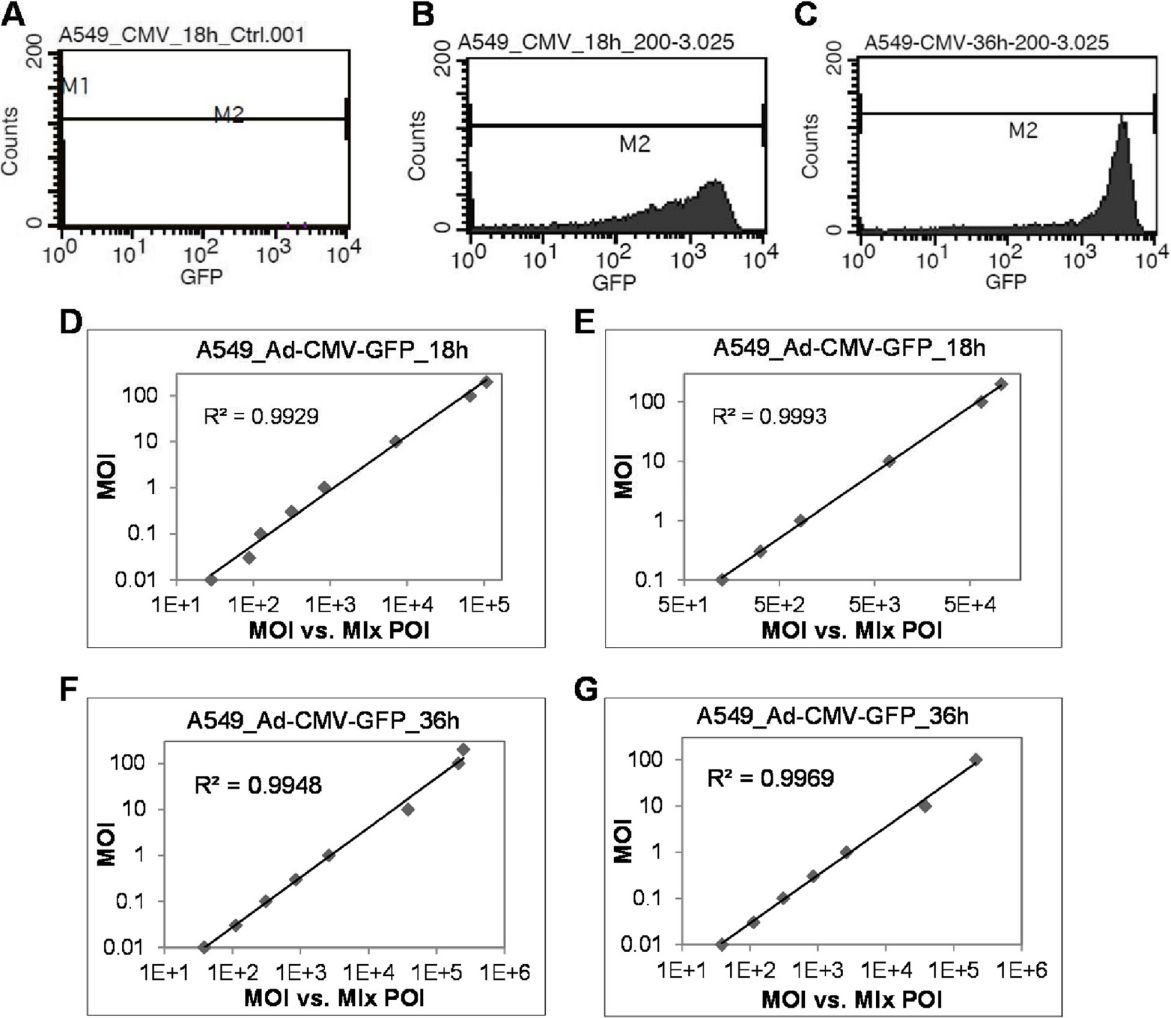
Correlation between MFI × POI with MOI of Ad-CMV-GFP in A549 cells with flow cytometer adjusted. Transduction at MOI 0.01 to 200 for 18 and 36 h. The sensitivity of the flow cytometer was adjusted to prevent the overshooting of a strong GFP signal. **(A-C)** GFP signals and cell count of control cells **(A)** and cells infected for 18 and 36 h **(B and C)**.** (D-G) **Correlation between MFI × POI and MOI. R^2^, coefficient of determination.

To extend the detection limitation, the sensitivity of the flow cytometer was adjusted lower to prevent the GFP signal from overshooting. A549 cells were transduced with Ad-CMV-GFP for 18 and 36 h (Figure 3A-C). With this adjustment, the MOI range for a good linear correlation was extended from 0.01 to 10 with the default setting to 0.01 to 200 (R^2^ = 0.993) at 18 hpi (Figure 3D vs Figure 2F). Similar to the result in Figure 2A, results at MOI 0.01 and 0.03 partially deviated from linearity, which may be caused by background signals interfering in the low GFP signal. A better linear correlation was detected at MOI 0.1 to 200 (R^2^ = 0.999) (Figure 3E). Moreover, the detection sensitivity was further adjusted for the data at 36 hpi in that the MOI range for a good linear correlation was extended from 0.01 to 1 with the default setting to 0.01 to 200 (R^2^ = 0.995) (Figure 3F vs Figure 2H). However, we detected a partial deviation of the data for 200 MOI, and better linearity was detected at MOI 0.01 to 100 (R^2^ = 0.997) (Figure 3G). This deviation may be caused by saturation of the protein expression machinery because of no overshooting of the GFP signal observed (Figure 3C).

### Correlation between average GFP intensity and MOI of Ad-CMV-GFP in H1299 cells

**Figure 4:**
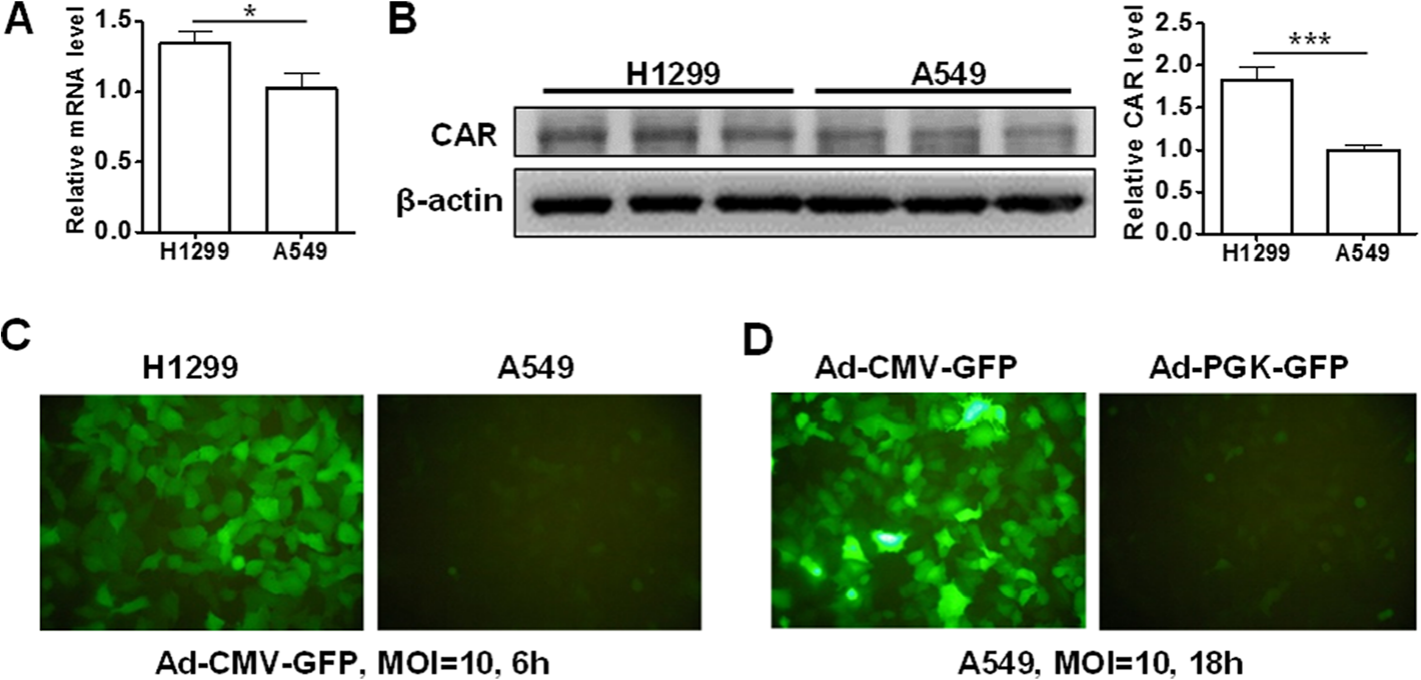
GFP signal with Ad-CMV-GFP or Ad-PGK-GFP transduction in A549 and H1299 cells. **(A)** Real-time RT-PCR of mRNA level and **(B)** western blot analysis of protein level of coxsackie and adenovirus receptor (CAR) in H1299 and A549 cells. Fluorescence microscopy of GFP in **(C)** H1299 and A549 cells with Ad-CMV-GFP transduction at MOI 10 for 6 h and **(D)** A549 cells with Ad-CMV-GFP and Ad-PGK-GFP transduction at MOI 10 for 18 h. Data are mean ± SEM (n = 6). *P < 0.05 and ***P < 0.001.

**Figure 5:**
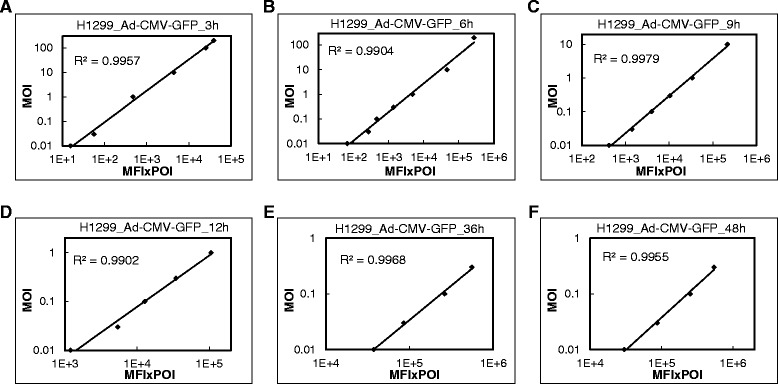
Correlation between MFI × POI and MOI of Ad-CMV-GFP in H1299 cells. Transduction at MOI 0.01 to 200 for 3 to 48 h for flow cytometry. **(A**-**F)** Correlation between MOI and MFI × POI. R^2^, coefficient of determination.

The coxsackie and adenovirus receptor determines adenovirus susceptibility [11,12]. We investigated the correlation between MFI × POI and MOI in H1299 cells, which showed higher coxsackie and adenovirus receptor expression at mRNA and protein levels (Figure 4A and B) and greater GFP level than A549 cells after Ad-CMV-GFP transduction (Figure 4C). We detected a good linear correlation at MOI 0.01 to 200 at 3 and 6 hpi (Figure 5A and B). Similar to results for A549 cells, MFI × POI deviated from linearity with high MOI and prolonged incubation (Additional file 1: Figure S2). The MOI ranges for good linearity was 0.01 to 10 at 9 hpi, 0.01 to 1 at 12, 18 and 24 hpi, and 0.01 to 0.3 at 24, 36 and 48 hpi (Figure 5C-F, Additional file 1: Figure S2).

### Correlation between average GFP intensity and MOI of Ad-PGK-GFP in A549 cells

**Figure 6:**
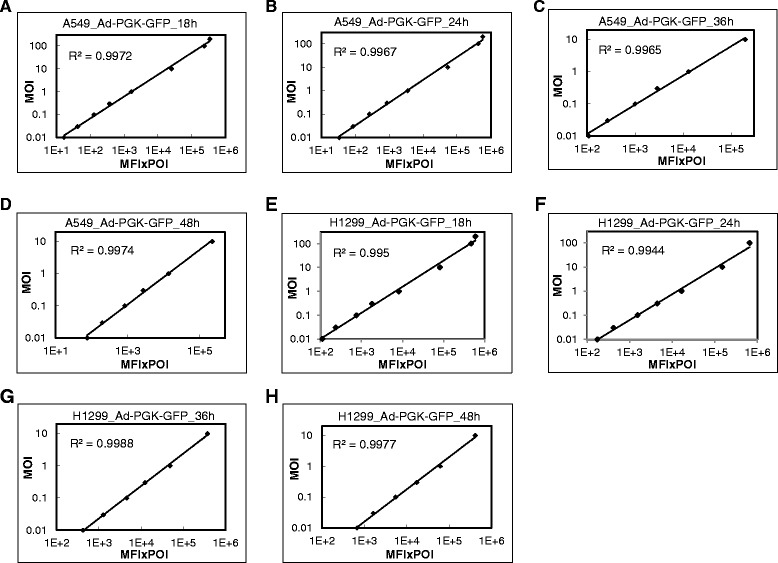
Correlation between MFI × POI and MOI of Ad-PGK-GFP in A549 and H1299 cells. A549 **(A-D)** and H1299 **(E-H)** cells infected at MOI 0.01 to 200 for 18 to 48 h. Correlation between MOI and MFI × POI. R^2^, coefficient of determination.

We next examined the linear correlation with GFP-expressing adenoviral vector driven by a weak promoter, Ad-PGK-GFP, in A549 cells. GFP level was low at MOI 10 as compared with Ad-CMV-GFP transduction at 18 hpi (Figure 4D). We detected good linear correlations at 18 and 24 hpi at MOI 0.01 to 200 (R^2^ = 0.997) (Figure 6A and B). Similarly, high MOI conferred deviation from linearity at 36 and 48 hpi (Additional file 1: Figure S3A-C). We detected better linear correlations at MOI 0.01 to 10 at 36 and 48 hpi (R^2^ = 0.997) (Figure 6C and D).

### Correlation between average GFP intensity and MOI of Ad-PGK-GFP in H1299 cells

In H1299 cells, we detected good linear correlation at MOI 0.01 to 200 at 18 hpi (R^2^ = 0.995) (Figure 6E). Similarly, high MOI conferred deviation from linearity at 24, 36 and 48 hpi (Additional file 1: Figure S3D-F). We detected good linear correlations at MOI 0.01 to 100 at 24 hpi and 0.01 to 10 at 36 and 48 hpi (Figure 6F-H).

### Correlation between POI and MOI and between MFI and MOI

**Figure 7:**
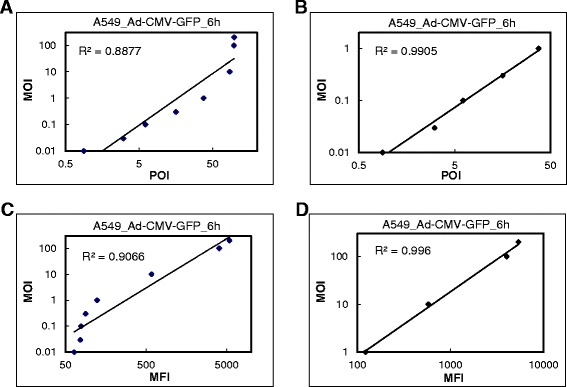
Correlation between MFI and MOI or between POI and MOI of Ad-CMV-GFP in A549 cells. Transduction at MOI 0.01 to 200 for 6 h as in Figure 2C. Correlation between POI and MOI **(A and B)** or MFI and MOI **(C and D)**. R^2^, coefficient of determination.

Under specific conditions, linear correlations between POI and MOI and between MFI and MOI have been reported [6,4]. To explore the correlation between POI and MOI or between MFI and MOI, we examined the results in A549 cells transduced with Ad-CMV-GFP for 6 h which showed a good linear correlation between MFI × POI and MOI 0.01 to 200 (Figure 2C). We found poor linear correlations between POI or MFI and MOI 0.01 to 200 (Figure 7A and C) but better linear correlations between POI and MOI 0.01 to 1 and between MFI and MOI 1 to 200 (Figure 7B and D).

### Comparison of CMV and PGK promoter activity for GFP expression

**Figure 8:**
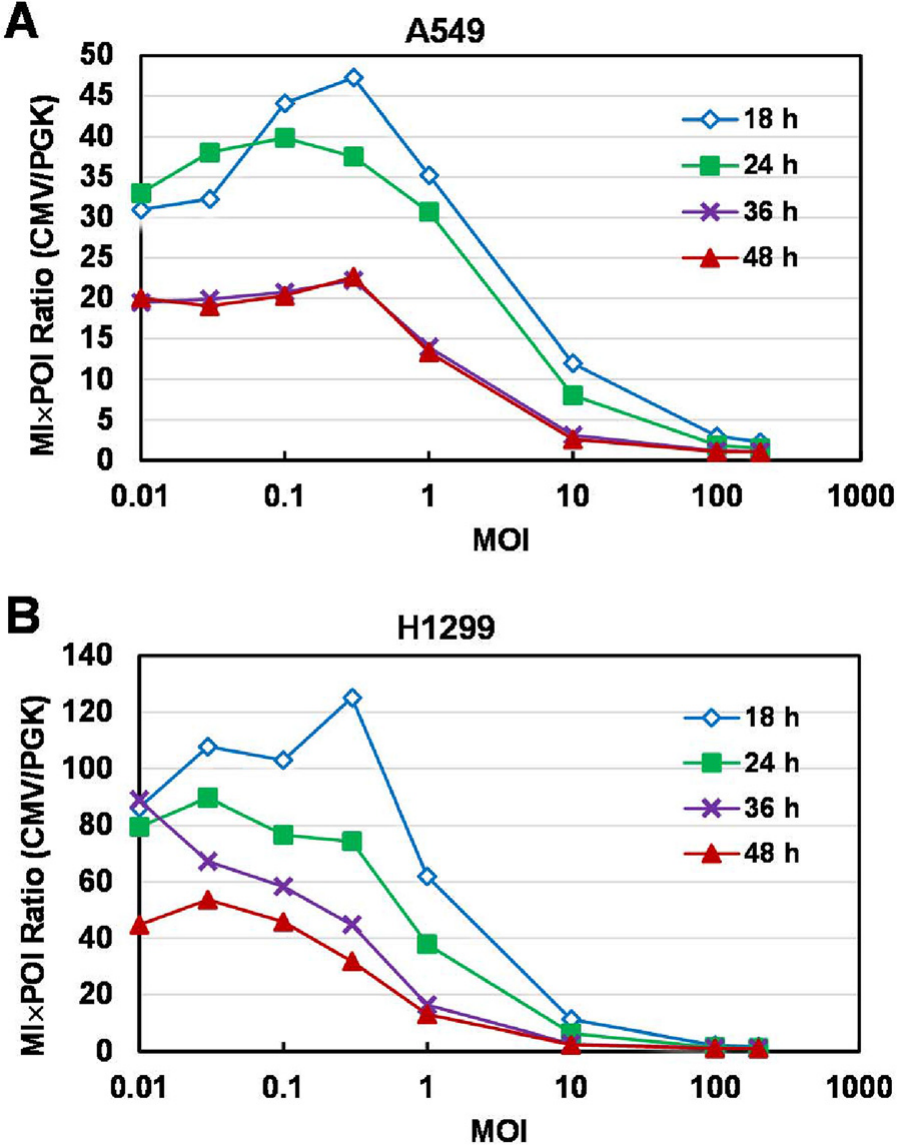
Comparison of CMV and PGK promoter for average GFP intensity. Ratio of MFI × POI with Ad-CMV-GFP and Ad-PGK-GFP transduction in A549 **(A)** and **(B)** H1299 cells.

Using MFI × POI to represent average GFP intensity, we compared the ratio of CMV and PGK promoter activity at MOI 0.1 and 0.3 which showed good linear correlations between MFI × POI and MOI among all time points. In A549 cells, the MFI × POI ratios were 20 to 47 times (Figure 8A). This ratio was decreased with longer incubation and higher MOI. Similar ratios were obtained, about 20 to 23 times, at 36 and 48 hpi. Moreover, we detected higher ratios in H1299 than A549 cells (Figure 8B). With longer incubation, this ratio decreased from > 100 times at 18 hpi to 32 times at 48 hpi. For both cell types, the ratio decreased rapidly at MOI ≥ 1. This finding may be caused by underestimation of the strong GFP signal which overshot the detection limitation of flow cytometer.

## Discussion

In this study, we examined the correlation of average GFP intensity with MOI of adenoviral vectors carrying GFP gene driven by the CMV and PGK promoters and observed a significant linear correlation between average GFP intensity and a wide range of MOI between 0.01 and 200, in transduced A549 and H1299 cells. Assessed by flow cytometry, the average GFP intensity was computed as MFI × POI. With the default flow cytometry settings, this linear correlation occurred at 3 to 6 hpi for the CMV promoter, whereas the linearity occurred at 18 to 24 hpi for the PGK promoter.

Because of the detection limitation of the flow cytometer for strong GFP signals, the linear correlation between MFI × POI and MOI deviated with high MOI and prolonged incubation. As compared with A549 cells, H1299 cells were infected by more adenovirus and therefore expressed a higher level of GFP, which resulted in a stronger signal and overshot the detection limitation of the flow cytometer at the same MOI. Our results reveal that decreasing the detection sensitivity of the flow cytometer prevents signal overshooting and extends the dynamic range for detecting strong GFP signals, which improves the linear correlation between MFI × POI and MOI. However, background signals may interfere with low GFP signals with decreased detection sensitivity.

A linear correlation between POI or MFI and MOI assessed by flow cytometry was previously proposed for viral titer estimation [4-6,13,14,7]. A good linear correlation between POI and MOI was found in cells infected with Ad-GFP at MOI < 0.23 or POI < 23% (R^2^ = 0.98) [6]. A method for virus titer estimation using flow cytometry suggested a linear correlation between amount of virus used and POI < 30% [15]. The limited range of MOI or POI may be due to the uneven infection of adenovirus in that cells do not receive the same number of virions. A low MOI or POI can avoid multiple infections by adenovirus. Moreover, gating cells with a similar degree of infection and the formulation of MFI of infected and non-infected cells produced a linear correlation between calculated results and MOI ranging from 5 to 100 [4]. These results are similar to our findings of a linear correlation between POI and MOI 0.01 to 1 and between MFI and MOI 1 to 200 in A549 cells with Ad-CMV-GFP transduction for 6 h (Figure 7). We further demonstrated a linear correlation between MFI × POI and a wide range of MOI. This correlation is limited by the detection limitation of the flow cytometer and the protein expression machinery but not the uneven infection of virions. This linear correlation could be used for titer estimation of adenovirus in a single day.

We compared CMV and PGK promoter activities with average GFP intensity and found that the ratios of CMV/PGK activities ranged from 20 to > 100. This result was supported by a previous report estimating the activity of the PGK promoter to be < 5% of that of the CMV promoter [8]. Moreover, the ratio of CMV to PGK promoter activity was higher in H1299 than A549 cells (Figure 8). At the same MOI, ratios of CMV to PGK activities varied between these two cell lines. As well, the interference of background signals and the overshooting of the strong GFP signal may affect this ratio. We therefore concluded that the ratio of promoter activity in the current study depends on cell type, incubation time and gene copy number.

## Conclusions

By using flow cytometry, we demonstrated a good linear correlation between average GFP intensity, calculated as MFI × POI, and a wide range of MOI, 0.01 to 200, in cells transduced by Ad-GFP vectors. This linear correlation is limited by the detection range of flow cytometer and the availability of host protein expression machinery and is affected by MOI, cell type and incubation time employed in the study. Moreover, the ratio of CMV to PGK promoter activity is governed by the same factors. This linear correlation may provide a rapid and convenient method to assess factors regulating protein expression and to estimate adenovirus titer.

## Additional file

Additional file 1:
**Figure S1 **Correlation between MFI × POI and MOI of Ad-CMV-GFP in A549 cells. **(A-F)** Transduction at MOI 0.01 to 200 for 9 to 48 h for flow cytometry. Correlation between MOI and MFI × POI. R^2^, coefficient of determination. **Figure S2** Correlation between MFI × POI and MOI of Ad-CMV-GFP in H1299 cells. **(A-I)** Transduction at MOI 0.01 to 200 for 9 to 48 h for flow cytometry. Correlation between MOI and MFI × POI. R^2^, coefficient of determination. **Figure S3** Correlation between MFI × POI and MOI of Ad-PGK-GFP in A549 and H1299 cells. A549 **(A-C)** and H1299 **(D-F)** cells infected at MOI 0.01 to 200 for 36 to 48 h for flow cytometry. Correlation between MOI and MFI × POI. R^2^, coefficient of determination.

## Abbreviations

MOI: Multiplicity of infection
GFP: Green fluorescent protein
MFI: Mean fluorescence intensity
POI: Percentage of infection
CMV: Cytomegalovirus
PGK: Phosphoglycerate kinase
