# Supplementary material for: Linear correlation between average fluorescence intensity of green fluorescent protein and the multiplicity of infection of recombinant adenovirus
Source: J Biomed Sci. 2015 May 14;22(1):31. doi: 10.1186/s12929-015-0137-z (PMC4430915; doi:10.1186/s12929-015-0137-z)
Supplement: Additional file 1: — Figure S1 Correlation between MFI × POI and MOI of Ad-CMV-GFP in A549 cells. (A-F) Transduction at MOI 0.01 to 200 for 9 to 48 h for flow cytometry. Correlation between MOI and MFI × POI. R2, coefficient of determination. Figure S2 Correlation between MFI × POI and MOI of Ad-CMV-GFP in H1299 cells. (A-I) Transduction at MOI 0.01 to 200 for 9 to 48 h for flow cytometry. Correlation between MOI and MFI × POI. R2, coefficient of determination. Figure S3 Correlation between MFI × POI and MOI of Ad-PGK-GFP in A549 and H1299 cells. A549 (A-C) and H1299 (D-F) cells infected at MOI 0.01 to 200 for 36 to 48 h for flow cytometry. Correlation between MOI and MFI × POI. R2, coefficient of determination. [file 12929_2015_137_MOESM1_ESM.docx]

**Linear correlation between average fluorescence intensity of green fluorescent protein and the multiplicity of infection of recombinant adenovirus**

Yi-Chen Tsai, Tsung-Huang Tsai, Chen-Ping Chang, Shu-Fen Chen, Yen-Ming Lee, and Song-Kun Shyue

**Online-Only Data Supplement**

**Figure S1**

**Figure S1.** **Correlation between MFI×POI and MOI of Ad-CMV-GFP in A549 cells.** **(A-F)** Transduction at MOI 0.01 to 200 for 9 to 48 h for flow cytometry. Correlation between MOI and MFI×POI. R^2^, coefficient of determination.

**Figure S2**

**Figure S2.** **Correlation between MFI×POI and MOI of Ad-CMV-GFP in H1299 cells.** **(A-I)** Transduction at MOI 0.01 to 200 for 9 to 48 h for flow cytometry. Correlation between MOI and MFI×POI. R^2^, coefficient of determination.

**Figure S3**

**Figure S3.** **Correlation between MFI×POI and MOI of Ad-PGK-GFP in A549 and H1299 cells.** A549 **(A-C)** and H1299 **(D-F)** cells infected at MOI 0.01 to 200 for 36 to 48 h for flow cytometry. Correlation between MOI and MFI×POI. R^2^, coefficient of determination.
